# Two-stage model of nanocone formation on a surface of elementary semiconductors by laser radiation

**DOI:** 10.1186/1556-276X-7-428

**Published:** 2012-07-31

**Authors:** Artur Medvid, Pavels Onufrijevs, Gatis Mozolevskis, Edvins Dauksta, Roberts Rimsa

**Affiliations:** 1 Institute of Technical Physics, Riga Technical University, Azenes iela 14/24, Riga LV-1048, Latvia; 2 Institute of Semiconductor Physics, NAS of Ukraine, 45 Prospekt Nauki, Kyiv 03028, Ukraine

**Keywords:** Laser radiation, P-n junction, Nanocones, Thermogradient effect

## Abstract

In this work, we study the mechanism of nanocone formation on a surface of elementary semiconductors by Nd:YAG laser radiation. Our previous investigations of SiGe and CdZnTe solid solutions have shown that nanocone formation mechanism is characterized by two stages. The first stage is characterized by formation of heterostructure, for example, Ge/Si heterostructure from SiGe solid solutions, and the second stage is characterized by formation of nanocones by mechanical plastic deformation of the compressed Ge layer on Si due to mismatch of Si and Ge crystalline lattices. The mechanism of nanocone formation for elementary semiconductors is not clear until now. Therefore, the main goal of our investigations is to study the stages of nanocone formation in elementary semiconductors. A new mechanism of p-n junction formation by laser radiation in the elementary semiconductor as a first stage of nanocone formation is proposed. We explain this effect by the following way: p-n junction is formed by generation and redistribution of intrinsic point defects in temperature gradient field – the thermogradient effect, which is caused by strongly absorbed laser radiation. According to the thermogradient effect, interstitial atoms drift towards the irradiated surface, but vacancies drift to the opposite direction – in the bulk of semiconductor. Since interstitials in Ge crystal are of n-type and vacancies are known to be of p-type, a n-p junction is formed. The mechanism is confirmed by the appearance of diode-like current–voltage characteristics after i-Ge irradiation crystal by laser radiation. The mechanism in Si is confirmed by conductivity type inversion and increased microhardness of Si crystal. The second stage of nanocone formation is laser heating up of top layer enriched by interstitial atoms with its further plastic deformation due to compressive stress caused by interstitials in the top layer and vacancies in the buried layer.

## Background

Nanostructures are the most investigated object in solid state physics, especially quantum confinement effect in quantum dots [1], quantum wires [2], and quantum wells [3]. Moreover, different shapes of nanostructures can lead to unique physical properties of material [4]. For example, nanocones, depending on a height of structure and solid angle α at top of it, can be quantum dots, quantum wires, or quantum wells [5,6]. The decrease of nanocone's solid angle α < 60° leads to fundamental changes of its properties. Quantum dot transforms into a quantum wire with gradually decreasing diameter from the base until the tip of the cone. This is a unique system which has wide technical applications, -for example, 1D-graded bandgap structure in elementary semiconductor is a photodetector with “bolometric” or selective type of photosensitivity, depending on irradiation side [7].

Our previous investigations have shown possibility to form cone-like nanostructures on a surface of elementary semiconductors – Ge [8], Si [9], and solid solutions SiGe [10] and CdZnTe [11]. According to our investigation, nanocone formation mechanism is characterised by two stages for SiGe and CdZnTe solid solutions [6]. The first stage is characterized by formation of heterostructure, for example, Ge/Si from SiGe or CdTe/CdZnTe from CdZnTe solid solutions, and the second stage is characterized by formation of nanocones due to mechanical plastic deformation of the compressed Ge layer on Si or CdTe on CdZnTe, respectively. Nevertheless, the mechanism of nanocone formation for elementary semiconductors is not clear until now. Therefore, the research was aimed to study the nanocone formation mechanisms in elementary semiconductors. As a result, a new mechanism of p-n junction formation by laser radiation in elementary intrinsic semiconductor as a first stage of the process is developed.

## Methods

In experiments i-Ge single crystals with resistivity ρ = 45 Ωcm; *N*_a_ = 7.4 × 10^11^ cm^-3^, *N*_d_ = 6.8 × 10^11^ cm^-3^, where *N*_a_ and *N*_d_ are acceptors’ and donors’ concentration respectively, and samples' size 16.0 × 3.0 × 2.0 mm^3^ was used. The samples were mechanically polished with diamond grease and chemically etched with H_2_O_2_ and CP-4 (HF:HNO_3_:CH_3_COOH in volume ratio 3:5:3). Commercial p- and n-type single crystal silicon substrates were investigated in the experiments as well. For the determination of conductivity type changes of Si wafer after irradiation by Nd: YAG laser radiation, Cu was electrochemically deposited from 5% CuSO_4_ solution on Si surface.

Different intensities and wavelengths of nanosecond Nd:YAG laser were used to irradiate the samples (pulse repetition rate 12.5 Hz, power *P* = 1.0 MW). The laser beam to the irradiated surface of the samples was directed normally. The diameter of the spot of the laser beam was 3 mm, and point-to-point method was used for irradiation of the samples. All experiments of nanocone formation were performed at atmospheric pressure, *T* = 20°C, and 60% humidity.

Current-voltage (I-V) characteristics were measured for the non-irradiated and irradiated samples. Measurements of I-V characteristics were performed by soldering 99% tin and 1% antimony alloy contacts directly on the irradiated surface of Ge and tin contacts on the opposite side. Measurements of I-V characteristics were done at room temperature and atmospheric pressure. Rectification ratio (RR) of I-V characteristics was used for the characterization of p-n junction.

The surface morphology was studied using atomic force microscope (AFM) and scanning electron microscope (SEM).

## Results and discussion

**Figure 1:**
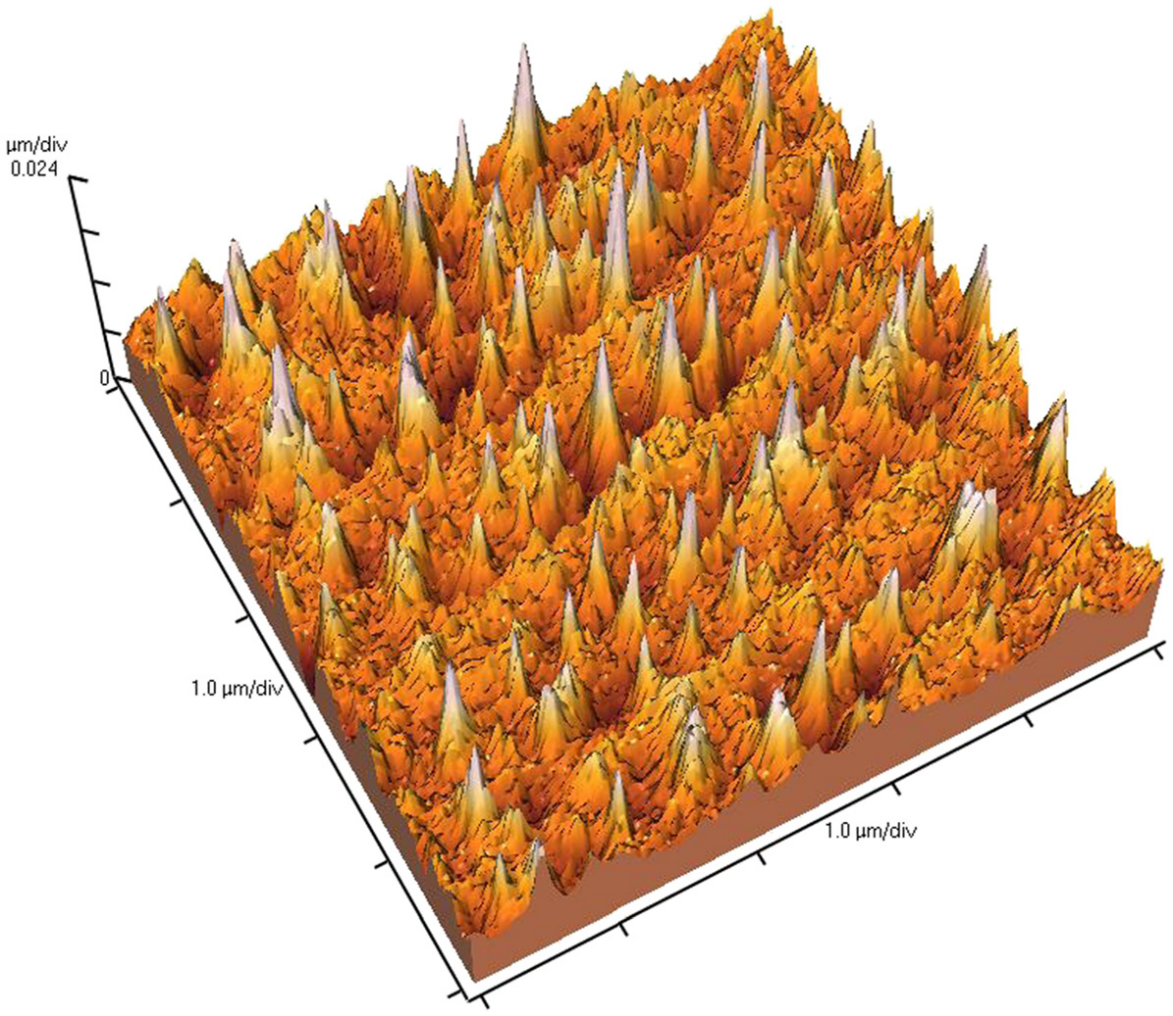
**AFM image of irradiated semiconductor surfaces.** 3D AFM image of Ge surface irradiated by Nd:YAG laser at intensity of 7.0 MW/cm^2^.

The results of irradiation of i-Ge single crystals by fundamental frequency (*λ*_1_ = 1,064 nm) of Nd:YAG laser at intensity higher than 7.0 MW/cm^2^ have shown possibility to form cone-like nanostructures, as shown in Figure 1. The ‘blueshifted’ photoluminescence spectrum obtained for Ge nanocones formed by laser radiation due to quantum confinement effect is shown in paper [8]. The ‘redshifted’ of LO phonon line with frequency 300 cm^-1^ by 6 cm^-1^ in Raman back scattering spectrum after irradiation by the laser is another good evidence of quantum confinement effect in nanocones formed by laser radiation [8].

**Figure 2:**
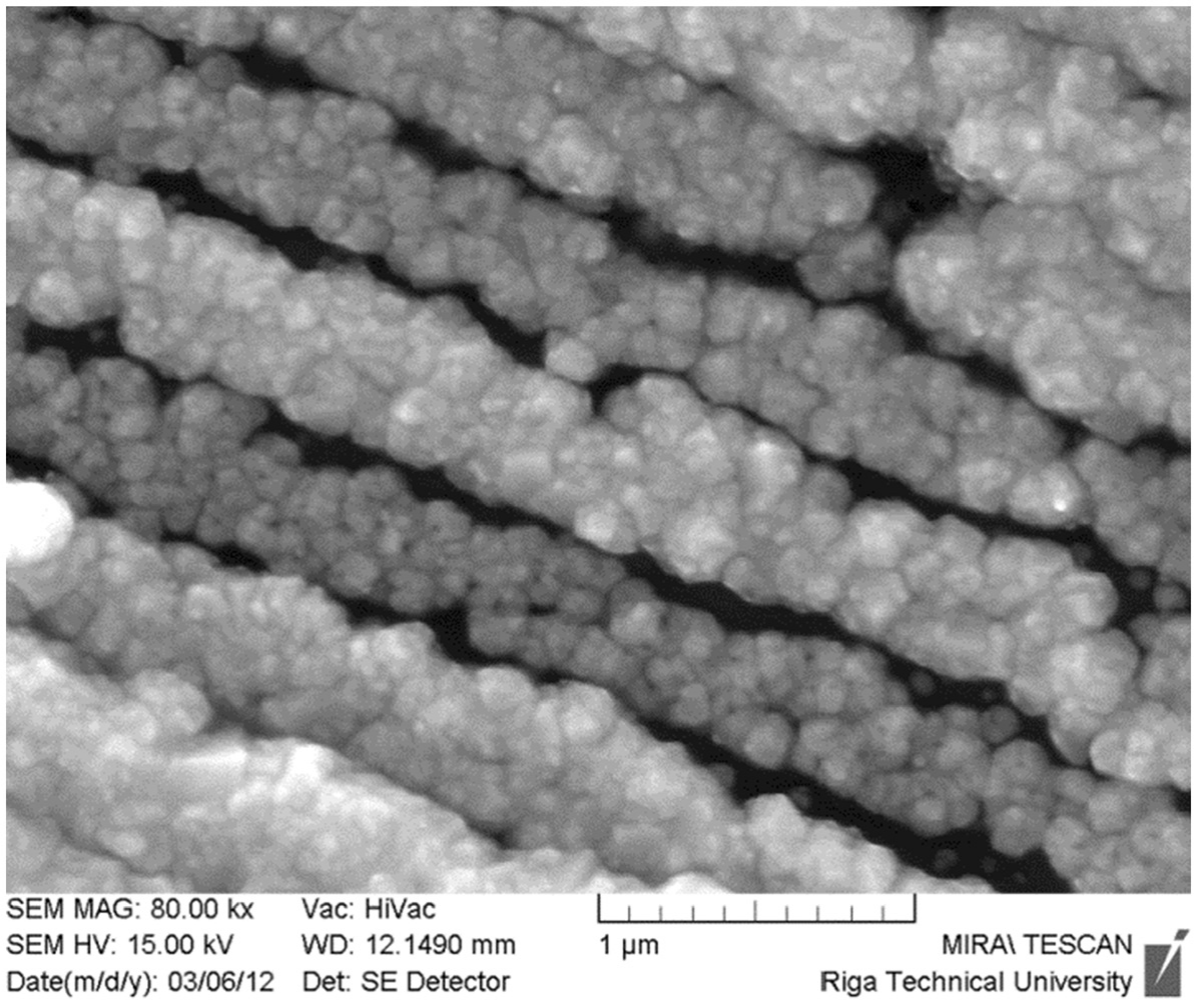
**SEM image of Si with deposited Cu.** SEM image of electrochemically deposited Cu lines on p-Si irradiated by Nd:YAG laser.

Investigation of the process of nanocone formation by different laser radiation intensities, using multimode laser, has shown a non-uniform deposition of Cu on p-Si substrates. After irradiation by multimode laser, laser-induced periodic surface structures are formed on the surface of Si. Cu was deposited on the top of periodic structures forming lines (see Figure 2).

It is known that Cu can be electrochemically deposited on n-type semiconductor [12]. Therefore, it was supposed that inversion of conductivity type takes place, and 'hills' of the periodic structures are of n-type. Possibility to invert conductivity type by laser radiation was shown in several p- and n-type semiconductors: p-Si [13-15], p-CdTe [16], p-InSb [17,18], p-InAs [19], p-PbSe [20], p-Ge [21], and n-HgCdTe [22]. Different mechanisms have been proposed to explain the nature of inversion of conductivity type, for example, impurities' segregation, defects' generation [20], amorphization [23] and oxygen-related donor generation [14]. However, there are many contradictions in the mechanisms. For example, n-type impurities in Si irradiated by laser cannot be oxygen atoms, according to paper [15]. Several authors have tried to explain p-n junction formation in n-type HgCdTe by defects’ generation, based on a model of defect formation related to interstitial mercury diffusion [24]. On the other hand, the authors of those papers did not take into account the effect of temperature gradient on the diffusion of atoms in solid solution. Moreover, it is theoretically shown that the p-n junction can be formed by redistribution of impurities in co-doped Si in gradient temperature field [25]. Thus, the mechanism of inversion of conductivity type by laser radiation is not clear until now.

**Figure 3:**
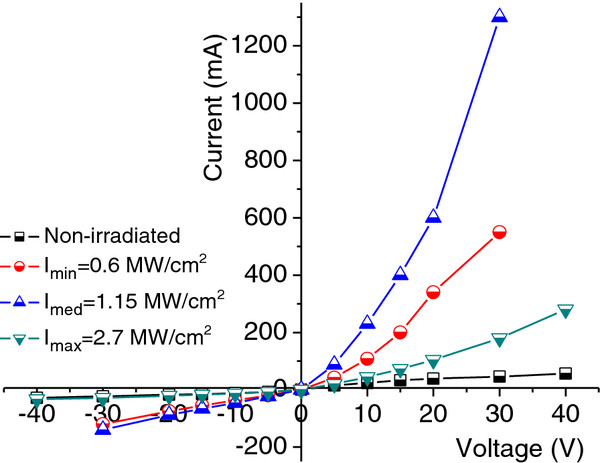
**Current**-**voltage characteristics of Ge samples.** Current-voltage characteristics of a non-irradiated and an irradiated i-Ge sample by Nd:YAG laser with different intensities at *λ* = 266 nm and 350 laser pulses.

**Figure 4:**
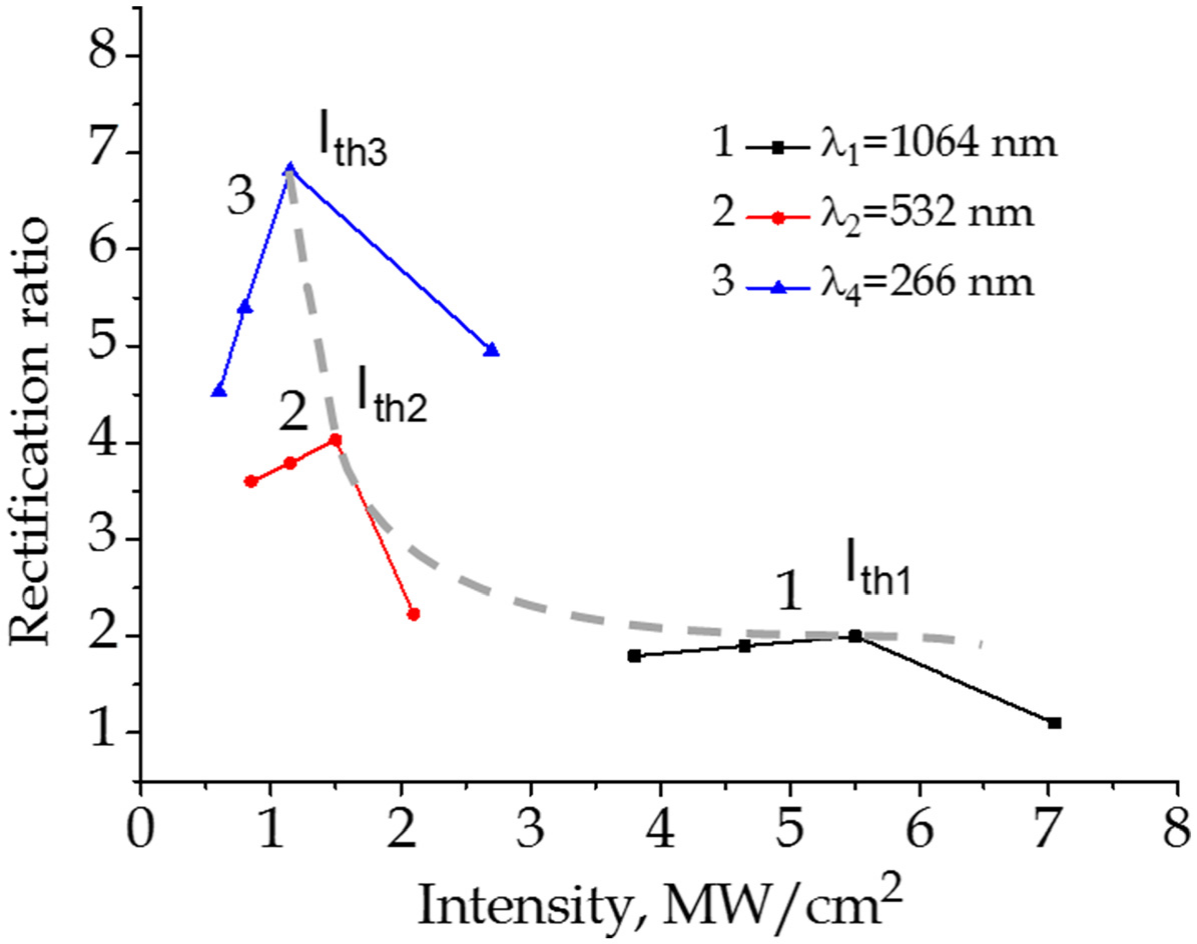
**Rectification ratio for p**-**n junctions formed by different laser parameters.** Rectification ratio as a function of irradiation intensity of Nd:YAG laser for different wavelengths of the laser radiation and 350 laser pulses.

**Figure 5:**
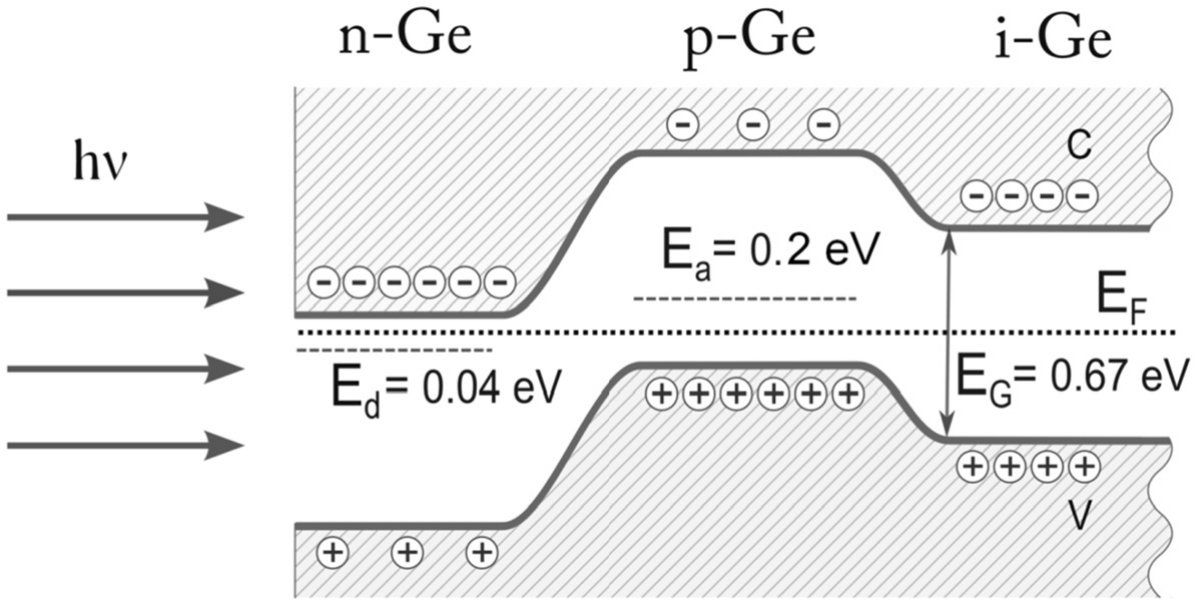
**Schematic illustration of n**-**p**-**i structure.** Schematic illustration of n-p-i structure formed by Nd:YAG laser radiation on the surface of i-Ge crystal.

I-V characteristics of i-Ge samples before and after irradiation by Nd:YAG laser with a wavelength of 266 nm and different laser intensities are shown in Figure 3. The I-V characteristic of the non-irradiated sample is linear. It means, I-V characteristics obey Ohm’s law, and therefore, there are no potential barrier between the electric contacts and the sample. After irradiation by the laser, I-V characteristics become diode like. Moreover, this process takes place in threshold manner, it means, RR is non-monotonic function on laser radiation intensity. These results are explained by damage of p-n junction at threshold intensity (*I*_th_) due to formation of nanocones by laser radiation on a surface of semiconductor. A more detailed investigation of RR as a function of irradiation intensity of Nd:YAG laser for different wavelengths of the laser radiation and 350 laser pulses is shown in Figure 4. Threshold intensities are observed at the fundamental frequency *I*_th1_ = 5.5 MW/cm^2^, the second harmonic *I*_th2_ = 1.5 MW/cm^2^, and the fourth harmonic *I*_th4_ = 1.15 MW/cm^2^, as shown in Figure 4 - the dashed line. We can see that RR and *I*_th_ are decreasing with increase of laser intensity and laser wavelength. We explain such behavior of I-V characteristics by formation of p-n junction at different depths which depends on penetration depth of laser beam. The decrease of the threshold intensity with increase of wavelength of Nd:YAG laser radiation and appearance of current-voltage characteristic rectification effect are explained in the following way: p-n junction is formed as a result of generation and redistribution of intrinsic point defects - vacancies and interstitial atoms in temperature gradient field - thermogradient effect [26], which is caused by strongly absorbed laser radiation. According to thermogradient effect, interstitial atoms drift towards the irradiated surface, but vacancies drift to the opposite direction - in the bulk of semiconductor. Since the interstitials in Ge crystal are of n-type and vacancies are known to be of p-type [27], a p-n junction is formed. Schematic illustration of n-p-i structure after irradiation of i-Ge sample is shown in Figure 5. We can see that shallow donor levels in band diagram according to the paper [28] has *E*_d_ = 0.04 eV, and deep acceptor level has *E*_a_ = 0.2 eV. The presence of deep acceptor level could be the main reason of low RR for p-n junction formed by laser radiation. For the improvement of I-V characteristics, it is necessary to use an acceptor with shallow level, for example Sb.

The mechanism of p-n junction formation by laser radiation in elementary semiconductors is the first stage of nanocone formation.

The second stage of nanocone formation in elementary semiconductors is laser heating up of the top layer enriched by interstitial atoms with further plastic deformation due to compressive stress caused by interstitials in the top layer and vacancies in the buried layer.

Additional evidence of two-stage mechanism for elementary semiconductors is non-monotonous dependence of microhardness of Si crystal as a function of the laser intensity [6,29] and compressive stress can be introduced in Si-SiO_2_ system by laser radiation [30].

## Conclusions

For the first time, a new mechanism of p-n junction formation in the elementary intrinsic semiconductor by laser radiation as the first stage of nanocone formation is proposed. P-n junction is formed by generation and redistribution of intrinsic point defects in temperature gradient field - the thermogradient effect, which is caused by strongly absorbed laser radiation. The second stage of nanocone formation is laser heating up of top layer enriched by interstitial atoms with its further plastic deformation due to compressive stress caused by interstitials in the top layer and vacancies in the buried layer.

## Abbreviations

AFM: atomic force microscope; I_th_: threshold intensity; I-V: current–voltage; RR: rectification ratio; SEM: scanning electron microscope.

## Competing interests

The authors declare that they have no competing interests.

## Authors’ contributions

AM conceived the studies and coordinated the experiment. All of the authors participated to the analysis of the data and wrote the article. PO, GM and ED carried out the sample preparation and the measurements for elementary semiconductors: Si, Ge.RR carried out the measurements of current–voltage characteristics. All authors read and approved the final manuscript.

## Authors’ information

Prof. Dr.habil.phys. AM is head of Semiconductors Laboratory at Riga Technical University. Dr.phys. PO is lead researcher in Semiconducor laboratory. ED and GM are PhD students under AM in Riga Technical University. RR is bachelor student in University of Leeds.
